# Disarming *Pseudomonas aeruginosa*: repurposing vardenafil, valsartan, and olmesartan as anti-virulence and antibiofilm agents

**DOI:** 10.1038/s41598-026-64238-9

**Published:** 2026-08-07

**Authors:** Aya A. Mahfouz, Hisham A. Abbas, Nehal Yousef, Abdelaziz Elgaml

**Affiliations:** 1 Department of Microbiology and Immunology, Faculty of Pharmacy, Zagazig University, Zagazig, 44519 Egypt; 2 Department of Microbiology and Immunology, Faculty of Pharmacy, Horus University-Egypt, New Damietta, 34518 Egypt; 3 Department of Microbiology and Immunology, Faculty of Pharmacy, Mansoura University, Mansoura, 35516 Egypt

**Keywords:** Vardenafil, valsartan, olmesartan, *Pseudomonas aeruginosa*, Anti-virulence, Antibiofilm, Drug discovery, Microbiology

## Abstract

The rapid emergence of antibiotic resistance represents a critical global health challenge, particularly in biofilm-associated infections caused by *Pseudomonas aeruginosa* (*P. aeruginosa*). Quorum sensing plays a pivotal role in regulating bacterial virulence and biofilm formation. Consequently, targeting quorum sensing-regulated virulence factors represents a promising anti-virulence strategy to combat antibiotic resistance. In this study, the FDA-approved drugs vardenafil, valsartan, and olmesartan did not significantly affect the growth of ten clinical *P. aeruginosa* isolates. At sub-inhibitory concentrations, these drugs significantly reduced the production of key virulence factors, including hemolysin, proteases, pyocyanin, lipases, as well as impairing swarming motility, and biofilm formation. Gene expression analysis confirmed marked downregulation of quorum sensing–associated genes in five selected isolates. In vivo experiments revealed a substantial reduction in bacterial pathogenicity following treatment. Molecular docking analyses further supported these findings, revealing favorable interactions of the tested drugs with key quorum sensing regulatory proteins. Collectively, these results highlight the anti-quorum sensing, anti-virulence, and antibiofilm potential of these repurposed drugs and suggest their possible application in topical formulations as adjunctive therapies to conventional antibiotics for managing severe pseudomonal infections.

**Supplementary Information:**

The online version contains supplementary material available at 10.1038/s41598-026-64238-9.

## Introduction

*Pseudomonas aeruginosa* is a Gram-negative opportunistic nosocomial pathogen that primarily infects immunocompromised patients^1^. It is a ubiquitous pathogen widely distributed in the environment and is classified among the ESKAPE group of clinically significant antimicrobial-resistant pathogens, which includes *Pseudomonas aeruginosa*,* Acinetobacter baumannii*,* Enterococcus faecium*,* Staphylococcus aureus*,* Klebsiella pneumoniae*, and *Enterobacter* spp^2^. *P. aeruginosa* represents the second most commonly isolated pathogen in intensive care unit settings, with transmission occurring predominantly via healthcare workers and colonized patients. Due to its remarkable adaptability and several virulence factors, *P. aeruginosa* is capable of infecting multiple body sites, leading to a wide spectrum of acute and chronic infections, including respiratory, ocular, and urinary tract infections, wound-associated sepsis, and meningitis^3^.

**Fig. 1:**
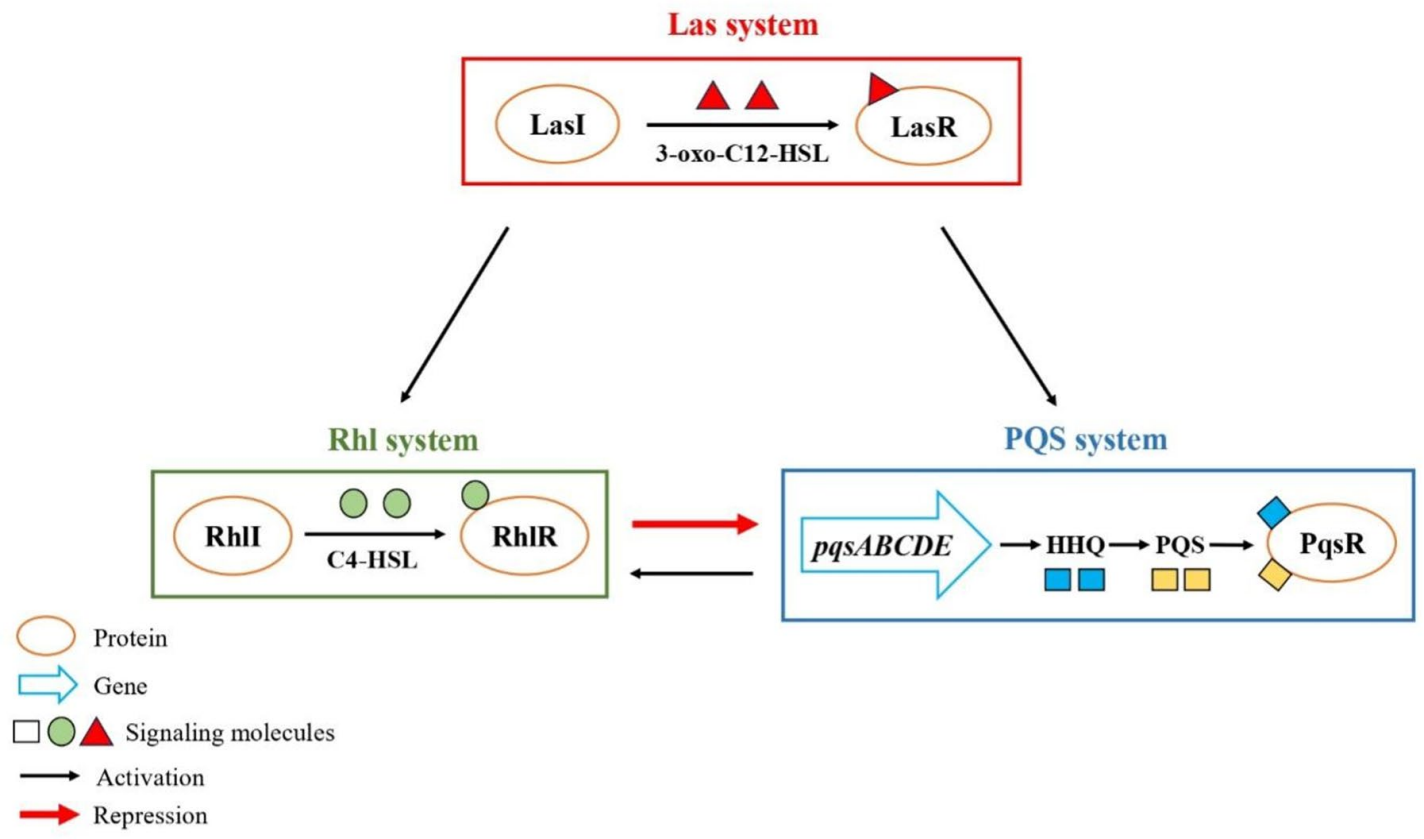
Schematic representation of the quorum sensing regulatory network in *Pseudomonas aeruginosa*. The diagram illustrates the hierarchical organization of the Las, PQS, and Rhl systems, including Las-dependent activation of PQS and Rhl signaling, PQS-mediated activation of the Rhl system, and Rhl-mediated repression of PQS signaling.

Bacterial communication is mediated by quorum sensing (QS), a regulatory system based on the production, secretion, and detection of small diffusible signaling molecules known as autoinducers (AIs)^4^. QS controls a wide range of bacterial behaviors, including virulence factor production, motility, biofilm formation, antibiotic resistance, and the secondary metabolites synthesis^5^. *P. aeruginosa* harbors three major QS systems, namely Las, PQS, and Rhl, which control virulence gene expression and coordinate intercellular communication, as shown in Fig. 1.

Interference with bacterial communication has emerged as a promising strategy for controlling the proliferation of pathogenic microorganisms^6^. Disruption of QS pathways can attenuate bacterial virulence, thereby reducing the bacterial pathogenicity and facilitating the removal by the host’s immune system. Several approaches have been explored to target the QS system of *P. aeruginosa*, the most prominent of which include^7^: (i) inhibition of autoinducer biosynthesis, (ii) prevention of AI binding to their receptors, and (iii) sequestration of AIs using antibodies or scavenger proteins.

QS inhibitors are being extensively investigated as preventive or adjunctive strategies for controlling antibiotic-resistant pathogens^8^. In this direction, the anti-virulence potential of several chemical moieties has been investigated, and FDA-approved drugs have gained interest^9^. Repurposing FDA-approved drugs represents a lower-risk and time-efficient strategy compared to *de novo* drug discovery, supported by their well-characterized pharmacokinetic and safety profiles^10^.

Vardenafil is a highly selective phosphodiesterase type 5 (PDE5) inhibitor that is used for treatment of erectile dysfunction^11^. The crystal structure studies show that vardenafil highly binds and competes for the catalytic domain of PDE5 and forms specific interactions with active-site residues, which explains its high inhibitory potency compared to sildenafil and tadalafil^12,13^. Additionally, vardenafil attenuated the antibacterial activity of ciprofloxacin when the two drugs were used in combination against *P. aeruginosa*^14^. Moreover, a previous study reported that sildenafil effectively inhibited biofilm formation, exhibited antimicrobial activity, and acted synergistically with commercially available antimicrobial agents to treat and prevent *P. aeruginosa* infections^15^.

Valsartan and olmesartan are widely known as angiotensin II receptor blockers (ARBs)^16^. They were selected as potential QS inhibitors due to their unique chemical scaffolds. Both valsartan and olmesartan contain a tetrazole ring along with additional functional groups; these structural features allow them to mimic certain hydrogen bonding and electrostatic interactions of natural QS autoinducers. Previous studies have demonstrated that tetrazole-containing compounds can act as effective QS modulators, by reducing biofilm formation and virulence factor production^17^. Moreover, telmisartan was reported to exhibit antimicrobial and antibiofilm potentials against many pathogens, including *P. aeruginosa*^18^. In addition, candesartan exhibited a synergistic antibacterial effect when co-administered with gentamicin and tobramycin against methicillin-resistant *S. aureus* (MRSA)^19^.

This study aims to evaluate the potential anti-virulence and antibiofilm activities of vardenafil, valsartan, and olmesartan against clinical isolates of *P. aeruginosa* in vitro and in vivo using phenotypic and genotypic techniques. Up to knowledge, this study provides the first evidence supporting the anti-virulence and antibiofilm effects of vardenafil, valsartan, and olmesartan against *P. aeruginosa*.

## Results

### The effect of vardenafil, valsartan, and olmesartan on *P. aeruginosa* growth

Prior to evaluating the anti-virulence effects of the tested agents, the potential influence of DMSO was assessed. The final DMSO concentration at the tested sub-inhibitory concentrations did not exceed 1% (v/v) for any of the tested drugs, and DMSO-treated controls showed no significant differences compared with untreated controls in any assay. These findings indicate that the observed effects were attributable to the tested compounds rather than to the solvent.

The ten highly virulent *P. aeruginosa* isolates exhibited swarming motility, hemolysin, protease, pyocyanin, and lipase production, as well as biofilm-forming ability. Vardenafil inhibited the growth of *P. aeruginosa* isolates at concentrations ranging from 3.12 to 6.25 mg/mL, whereas valsartan and olmesartan exhibited higher antimicrobial activity at lower concentrations (1.56–3.12 mg/mL) (**Supplementary Table ****S1****)**. Sub-inhibitory concentrations (1/8 minimum inhibitory concentrations (MIC)) used in all subsequent experiments ranged from 0.39 to 0.78 mg/mL for vardenafil and 0.2 to 0.39 mg/mL for valsartan and olmesartan. To eliminate potential effects of the tested drugs on bacterial growth, all anti-virulence activities were assessed using these sub-inhibitory concentrations.

**Fig. 2:**
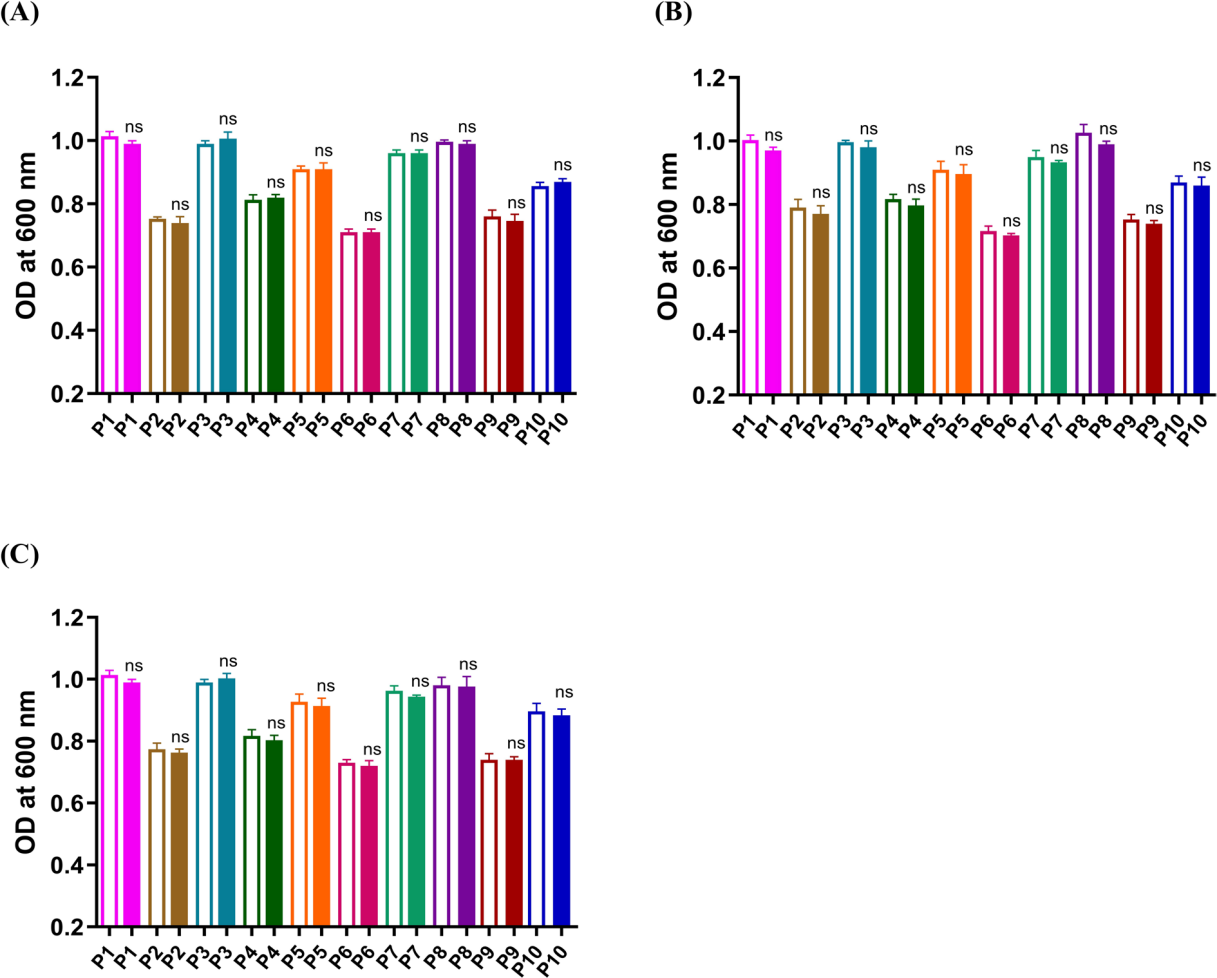
Effect of sub-inhibitory concentrations of repurposed drugs on the growth of *P. aeruginosa* isolates. The growth of the isolates did not differ significantly after adding **(A)** vardenafil, **(B)** valsartan, and **(C)** olmesartan compared with untreated isolates. Data are presented as mean ± standard deviation (SD). Empty bars represent untreated controls, whereas solid-colored bars represent drug-treated samples. **ns**: non-significant.

Additionally, the impact of the drugs on bacterial growth was evaluated by comparing the optical densities and viable counts of treated cultures with untreated and DMSO controls. Experiments were performed in triplicate, showing no significant effect of sub-MIC drug treatments on bacterial growth, as illustrated in Fig. 2.

### Vardenafil, valsartan, and olmesartan decreased hemolytic activity of *P. aeruginosa*

**Fig. 3:**
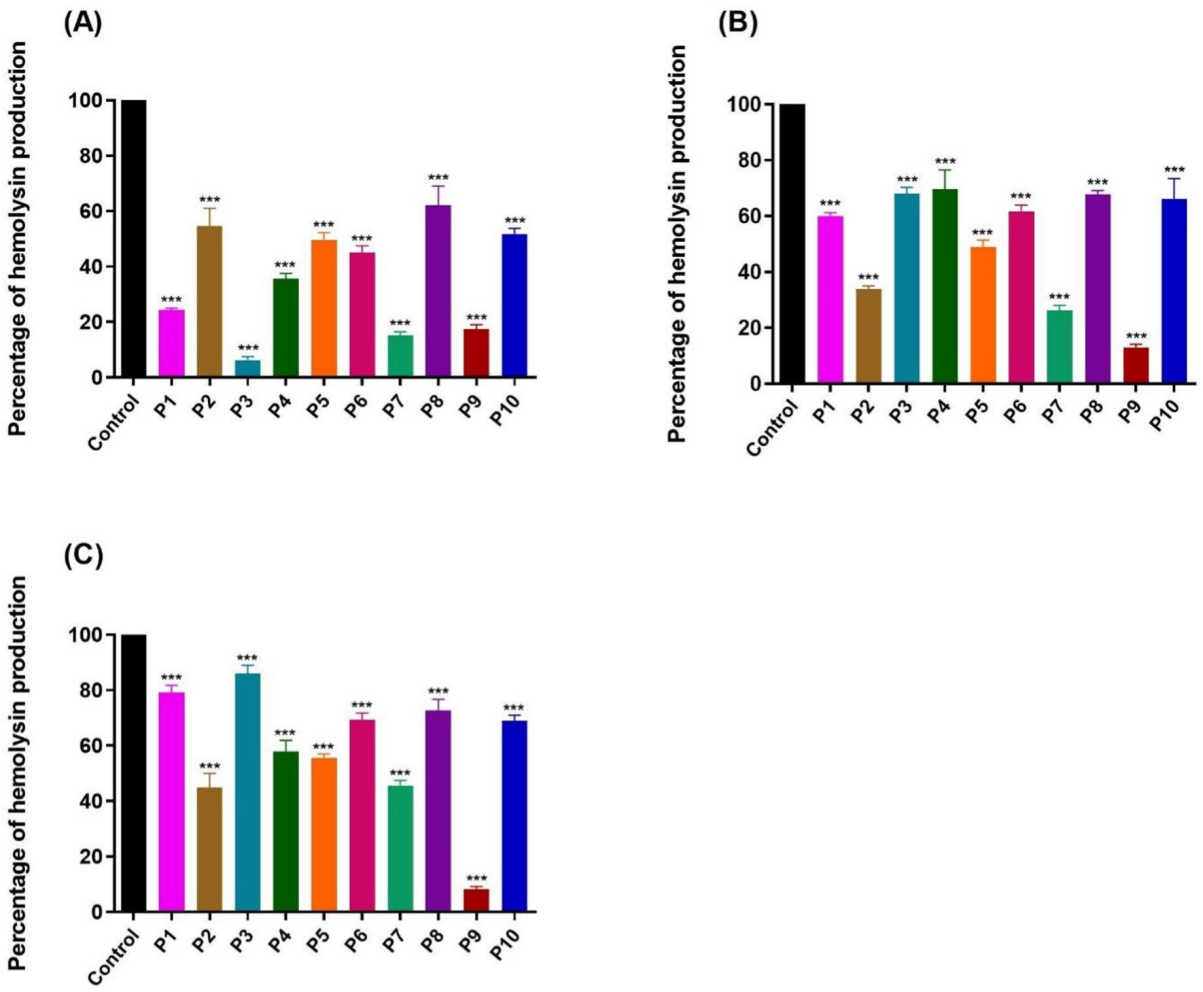
Inhibition of hemolysin production in *P. aeruginosa* isolates. Hemolysin activity following treatment with **(A)** vardenafil, **(B)** valsartan, and **(C)** olmesartan at 1/8 MIC compared with untreated isolates. The tested sub-inhibitory levels (1/8 MIC) ranged from 0.39 to 0.78 mg/mL for vardenafil and from 0.2 to 0.39 mg/mL for valsartan and olmesartan. Data are expressed as percentage change relative to the untreated control and presented as mean ± SD. ***p < 0.001.

The effect of the tested drugs on hemolytic activity was evaluated by comparing untreated *P. aeruginosa* isolates with drug-treated cultures and DMSO controls. Treatment with vardenafil, valsartan, and olmesartan resulted in a significant reduction in hemolysin activity compared to untreated cells. At sub-MIC concentrations, hemolysin activity was reduced by 37.97% to 93.75% for vardenafil, 30.1% to 87.02% for valsartan, and 13.97% to 91.63% for olmesartan **(**Fig. 3**).**

### Vardenafil, valsartan, and olmesartan reduced protease activity of *P. aeruginosa*

**Fig. 4:**
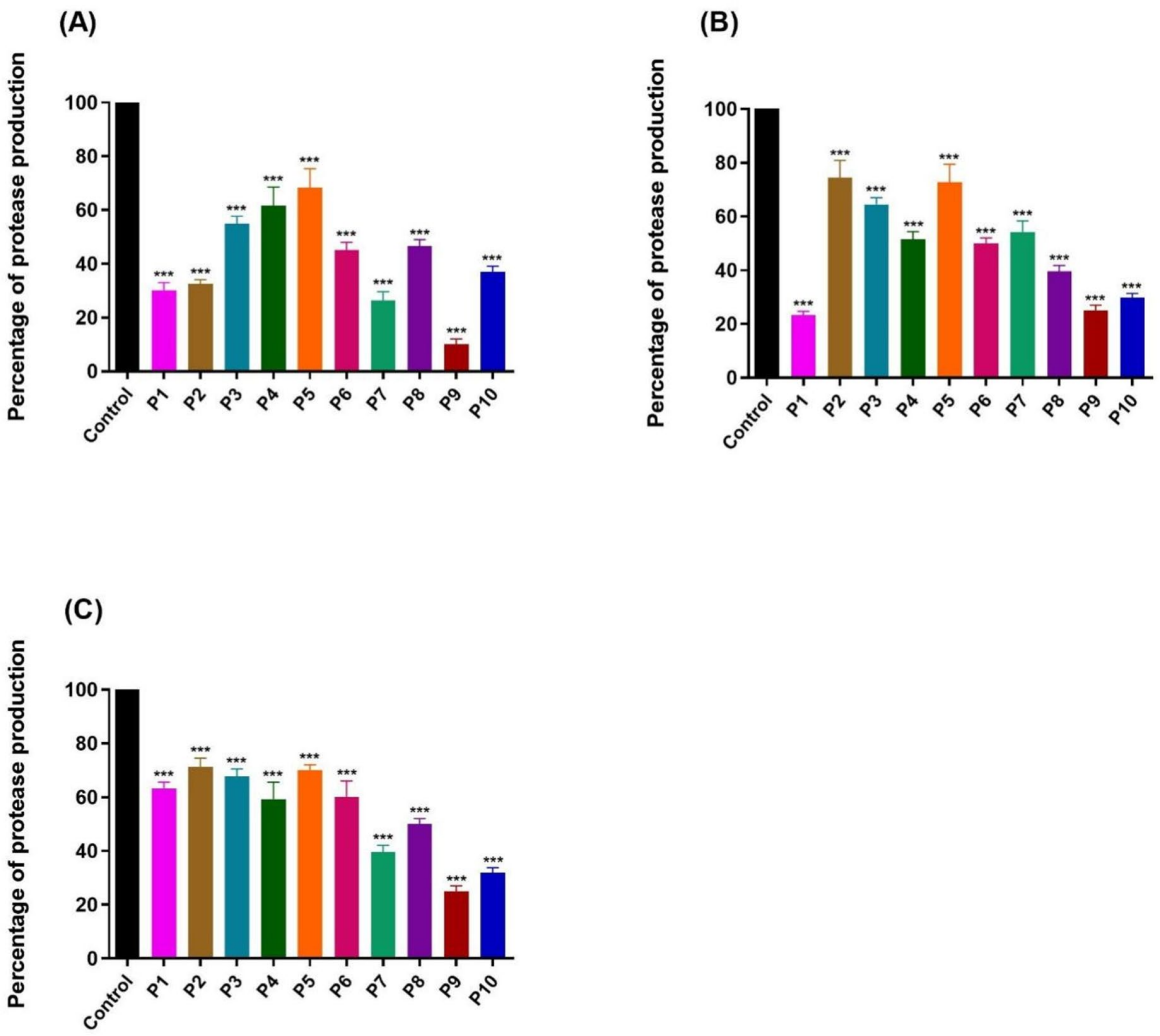
Inhibition of protease activity in *P. aeruginosa* isolates. Protease production upon treatment with **(A)** vardenafil, **(B)** valsartan, and **(C)** olmesartan when compared with untreated isolates. The tested compounds were used at 1/8 MIC, corresponding to 0.39–0.78 mg/mL for vardenafil and 0.2–0.39 mg/mL for valsartan and olmesartan. The results are expressed as percentage change from the untreated controls and presented as mean ± SD. ***p < 0.001.

Protease activity was quantitatively assessed using a modified skimmed milk broth assay. Treatment with vardenafil, valsartan, and olmesartan resulted in a marked reduction in protease production among the tested *P. aeruginosa* isolates, with inhibition ranging from 31.82% to 90% for vardenafil, from 25.58% to 76.67% for valsartan, and from 28.75% to 75% for olmesartan compared with untreated isolates and DMSO controls **(**Fig. 4**).**

### Pyocyanin activity was decreased after adding vardenafil, valsartan, and olmesartan

**Fig. 5:**
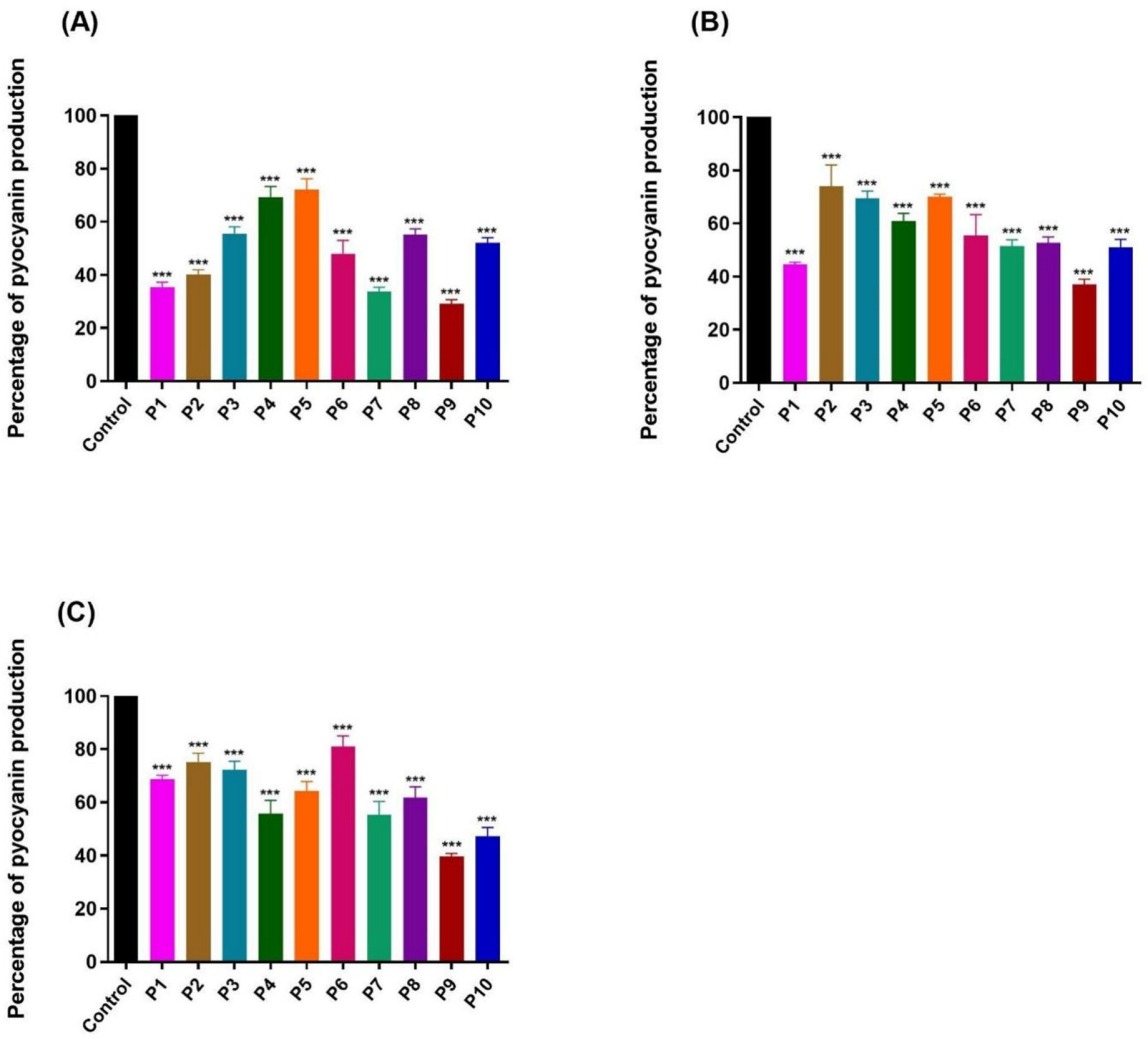
Reduction of pyocyanin production in *P. aeruginosa* isolates. Pyocyanin levels following treatment with **(A)** vardenafil, **(B)** valsartan, and **(C)** olmesartan compared with untreated isolates. The tested 1/8 MIC concentrations were 0.39–0.78 mg/mL for vardenafil and 0.2–0.39 mg/mL for valsartan and olmesartan. Values are reported as percent change with respect to untreated controls, with error bars representing SD. ***p < 0.001.

Pyocyanin production, a blue-green antioxidant pigment of *P. aeruginosa*, was spectrophotometrically assessed following treatment with the tested drugs at sub-MIC concentrations and compared with untreated cultures and DMSO controls. A reduction in pyocyanin production was observed among the 10 *P. aeruginosa* isolates, ranging from 27.77% to 70.89% with vardenafil, from 26% to 63% with valsartan, and from 22% to 60.33% with olmesartan **(**Fig. 5**)**.

### Lipase activity was decreased after treatment with vardenafil, valsartan, and olmesartan

**Fig. 6:**
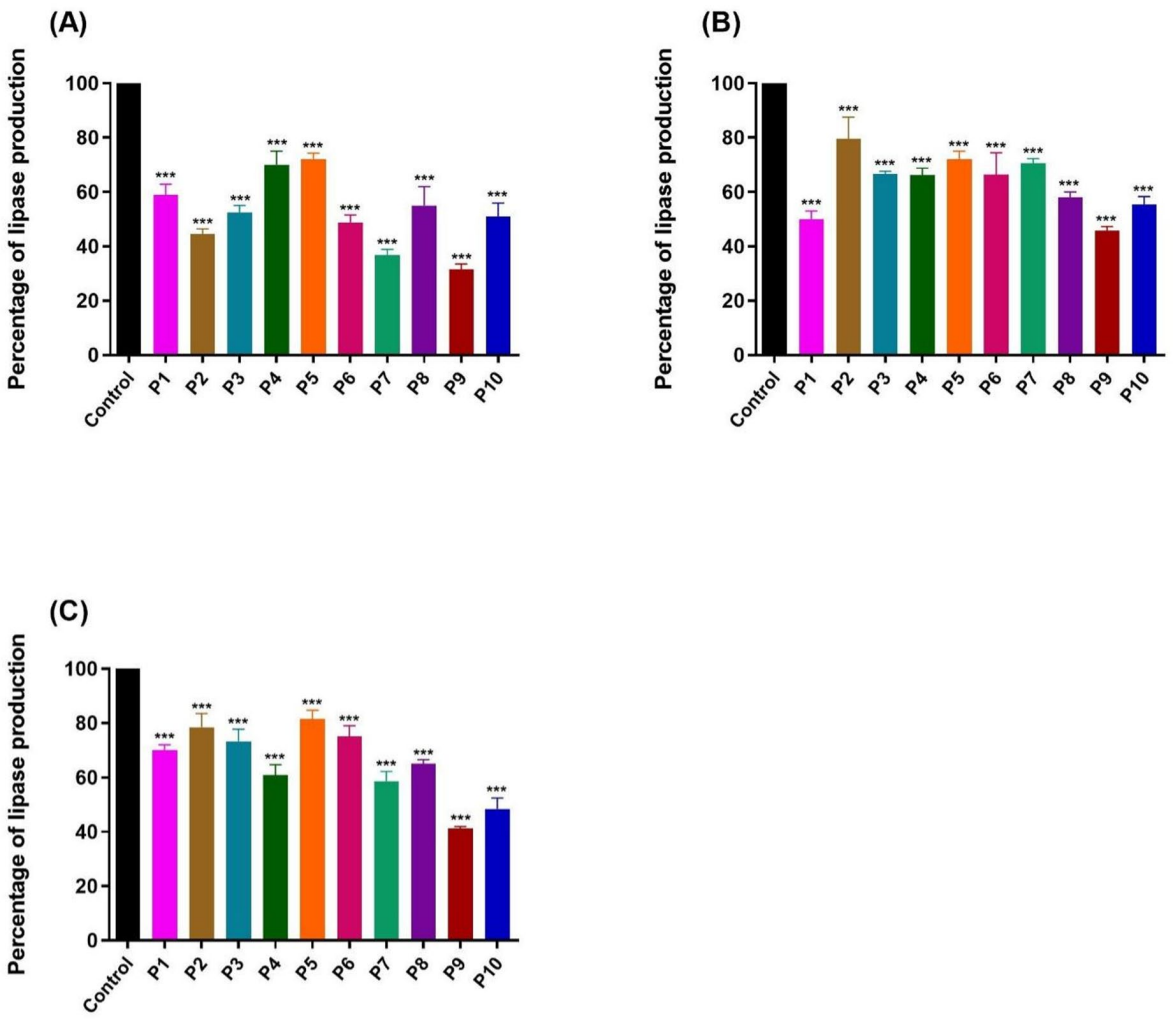
Inhibition of lipase activity in *P. aeruginosa* isolates. Lipase production after treatment with **(A)** vardenafil, **(B)** valsartan, and **(C)** olmesartan at their respective 1/8 MIC values. Data are shown as % change relative to untreated controls, and error bars indicate SD. ***p < 0.001.

Lipase activity was assessed using the Tween 80 opacity assay. Vardenafil treatment led to a reduction in lipase activity ranging from 27.88% to 68.48%, while valsartan caused inhibition ranging from 20.43% to 54.1% at 1/8 MIC. Additionally, olmesartan reduced lipase activity by 18.5% to 58.77% compared with untreated *P. aeruginosa* cultures and DMSO controls **(**Fig. 6**).**

### Inhibition of swarming motility by vardenafil, valsartan, and olmesartan

**Fig. 7:**
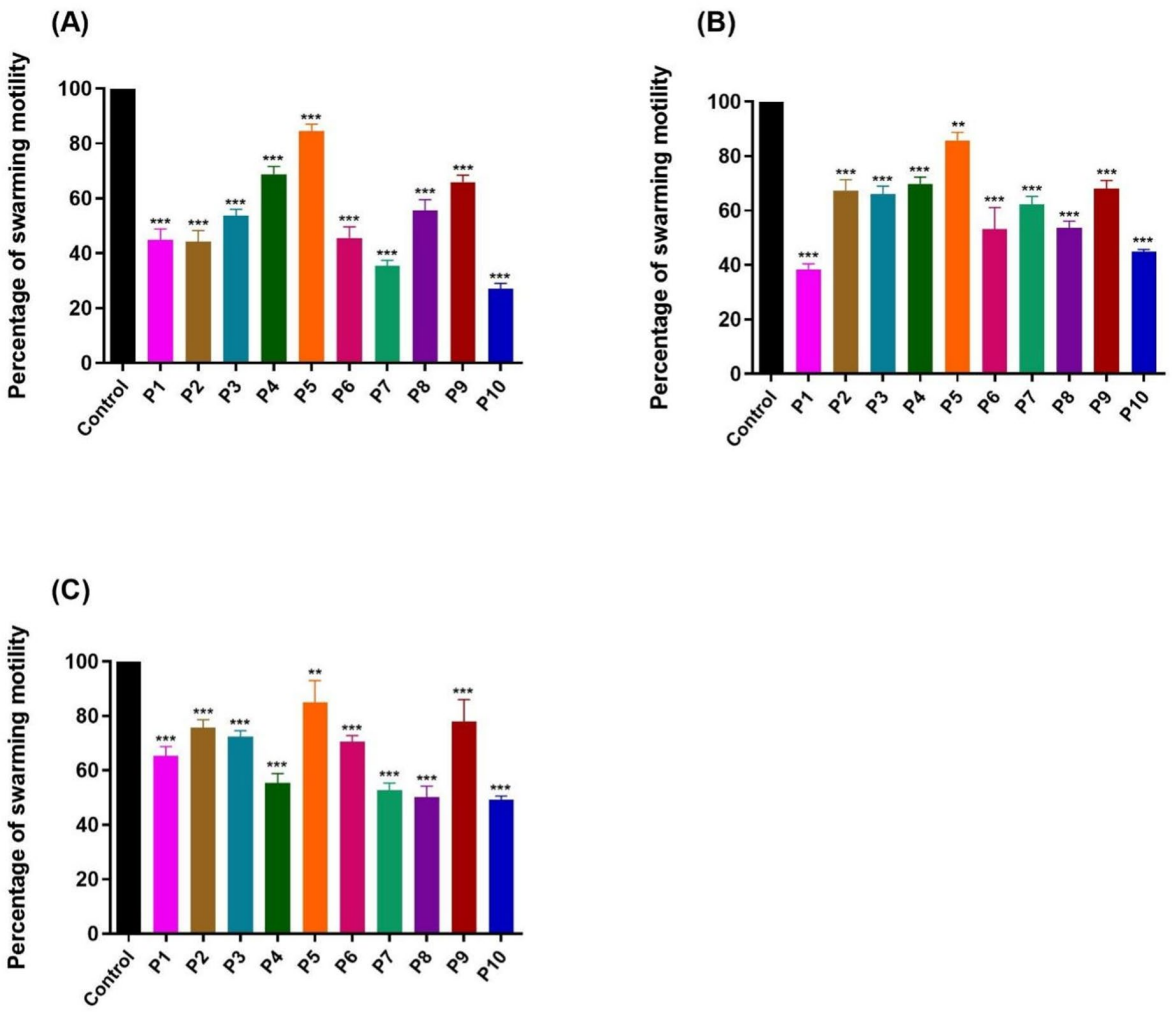
Attenuation of swarming motility in *P. aeruginosa* isolates. Swarming areas following treatment with **(A)** vardenafil, **(B)** valsartan, and **(C)** olmesartan at 1/8 MIC. The motility is expressed as a percentage relative to the untreated controls and presented as mean ± SD. **p < 0.01, ***p < 0.001.

Swarming motility of the 10 *P. aeruginosa* isolates was evaluated on swarming agar supplemented with the tested drugs at sub-MIC concentrations and compared with untreated and DMSO control plates. Vardenafil reduced the swarming ability of *P. aeruginosa* isolates by approximately 15.5% to 73%, whereas valsartan and olmesartan caused inhibition ranging from 14.17% to 61.77% and from 15% to 53%, respectively **(**Fig. 7**)**.

### Antibiofilm activities of vardenafil, valsartan, and olmesartan

**Fig. 8:**
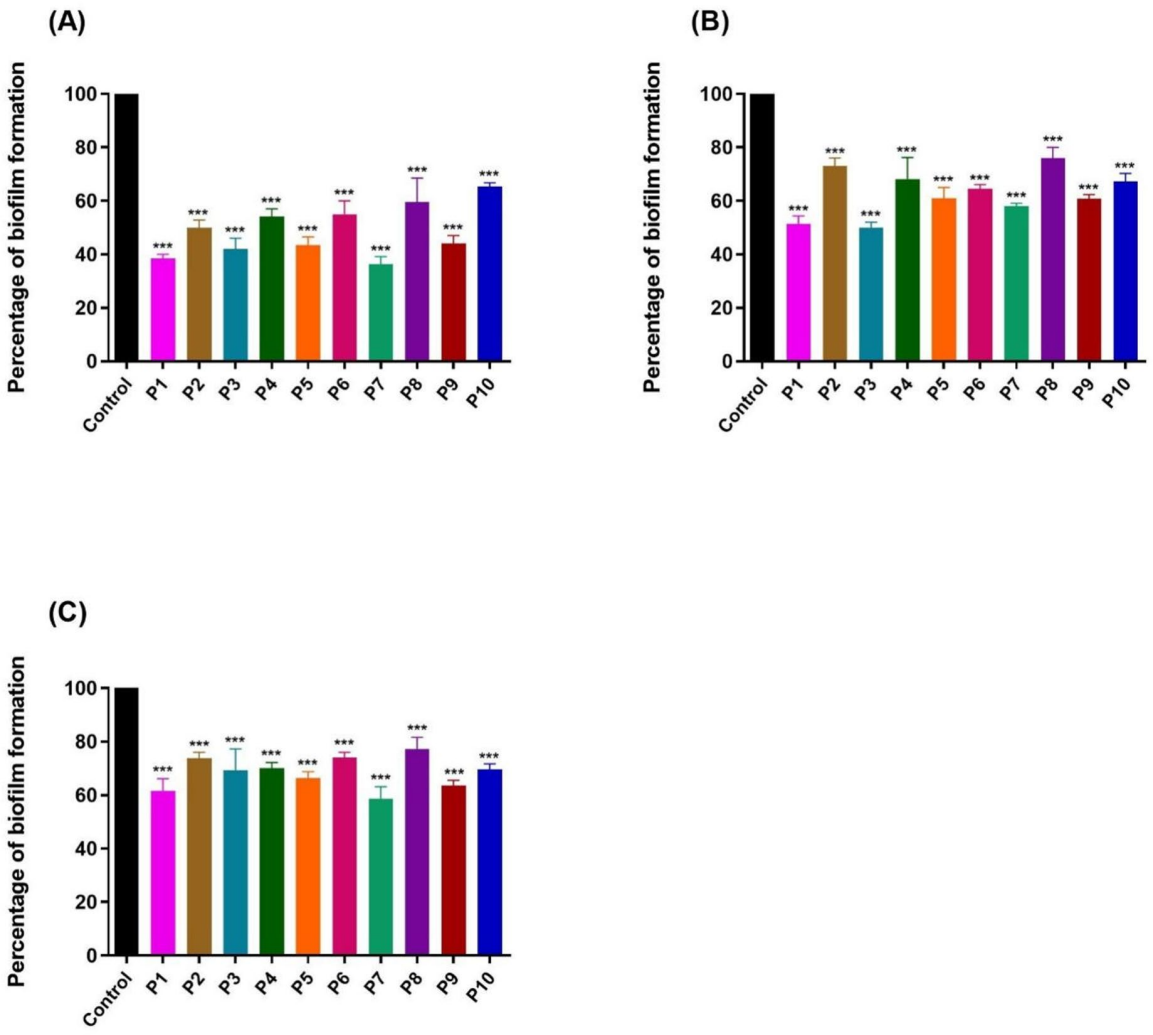
Inhibition of biofilm formation in *P. aeruginosa* isolates. Biofilm biomass following treatment with **(A)** vardenafil, **(B)** valsartan, and **(C)** olmesartan at sub-MICs. Results are presented as mean ± SD. ***p < 0.001.

The biofilm inhibitory activity of the tested drugs was assessed using the crystal violet spectrophotometric method. The sub-inhibitory concentrations of tested drugs reduced biofilm formation of ten *Pseudomonas* isolates. The percentage reduction ranged from 34.7% to 63.66% for vardenafil, 24% to 45.95% for valsartan, and 22.7% to 38.46% for olmesartan when compared with untreated isolates and DMSO controls **(**Fig. 8**).**

### Concentration-dependent anti-virulence activity of vardenafil

As vardenafil showed the strongest anti-virulence activity among the tested drugs, additional sub-MIC concentrations (1/4 and 1/2 MIC) were evaluated, alongside 1/8 MIC, against five representative *P. aeruginosa* isolates to investigate its concentration-dependent anti-virulence effects. As shown in **Supplementary Fig. ****S1**, vardenafil exhibited dose-dependent inhibition of the tested virulence factors, with greater inhibitory effects observed at higher sub-MIC concentrations.

### Downregulation of virulence and QS genes by vardenafil, valsartan, and olmesartan

**Fig. 9:**
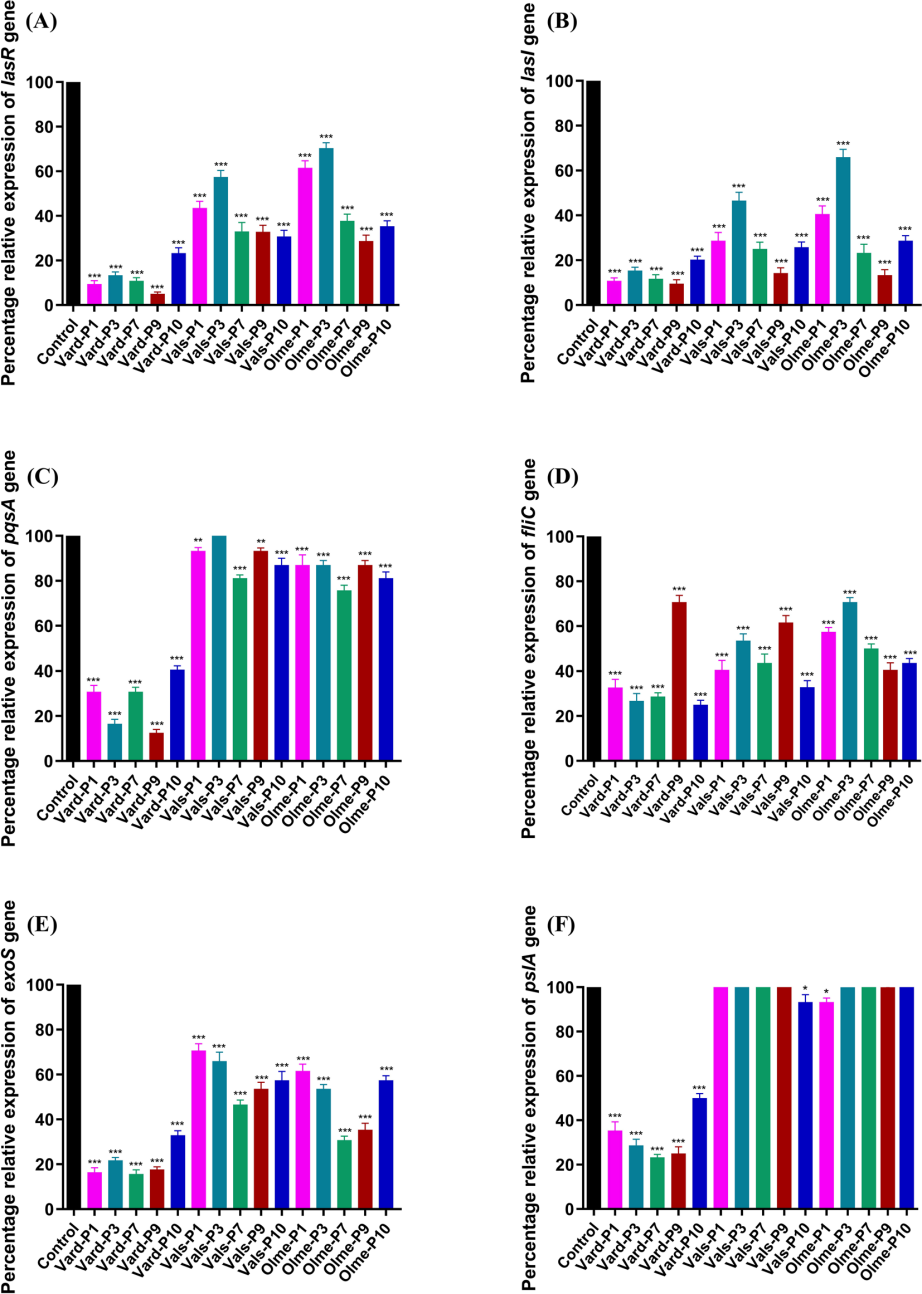
Downregulation of quorum sensing and virulence-related genes by repurposed drugs. Relative expression levels of **(A)** *lasR*, **(B)** *lasI*, **(C)** *pqsA*, **(D)** *fliC*, **(E)** *exoS*, and **(F)** *pslA* following treatment with vardenafil, valsartan, and olmesartan. The tested drugs were used at 1/8 MIC, corresponding to 0.39–0.78 mg/mL for vardenafil, 0.2–0.39 mg/mL for valsartan, and 0.2 mg/mL for olmesartan. Gene expression levels were normalized against the housekeeping gene *ropD*. *p < 0.05, **p < 0.01, ***p < 0.001.

Using the comparative threshold cycle (2⁻^ΔΔCt^) method, sub-MIC treatment with the tested drugs decreased the expression of *lasR*, *lasI*, *pqsA*, *fliC*, *exoS*, and *pslA* in five *P. aeruginosa* isolates. The tested drugs were used at 1/8 MIC, corresponding to 0.39–0.78 mg/mL for vardenafil, 0.2–0.39 mg/mL for valsartan, and 0.2 mg/mL for olmesartan. Among the tested drugs, vardenafil exhibited the most pronounced downregulatory effect on all tested genes across all isolates. Specifically, vardenafil significantly reduced the expression levels of *lasR*, *lasI*, *pqsA*, *fliC*, *exoS*, and *pslA* by ranges of 76.67–94.92%, 79.69–90.53%, 59.39–87.5%, 29.29–75%, 67.01–84.61%, and 50–76.67%, respectively **(**Fig. 9**).**

The *lasR* expression was further downregulated by valsartan (42.57–69.22%) and olmesartan (38.44–69.22%). The *lasI* expression level was reduced from 53.35 to 85.64% by valsartan and from 34.02 to 86.6% by olmesartan. Valsartan suppressed *fliC* and *exoS* genes expression by ranges of 38.44–67.01% and 29.29–53.35%, respectively, whereas olmesartan reduced *fliC* and *exoS* expression by 29.29–59.39% and 38.44–69.22%, respectively. In contrast, both valsartan and olmesartan showed a weak downregulatory activity against *pqsA* expression in the five tested isolates with inhibition ranges of 0–18.78% and 12.95–24.21%, respectively. On the other hand, both drugs didn’t influence the expression level of *pslA* gene **(**Fig. 9**).**

### Vardenafil, valsartan, and olmesartan attenuated *P. aeruginosa* pathogenicity  in vivo

**Fig. 10:**
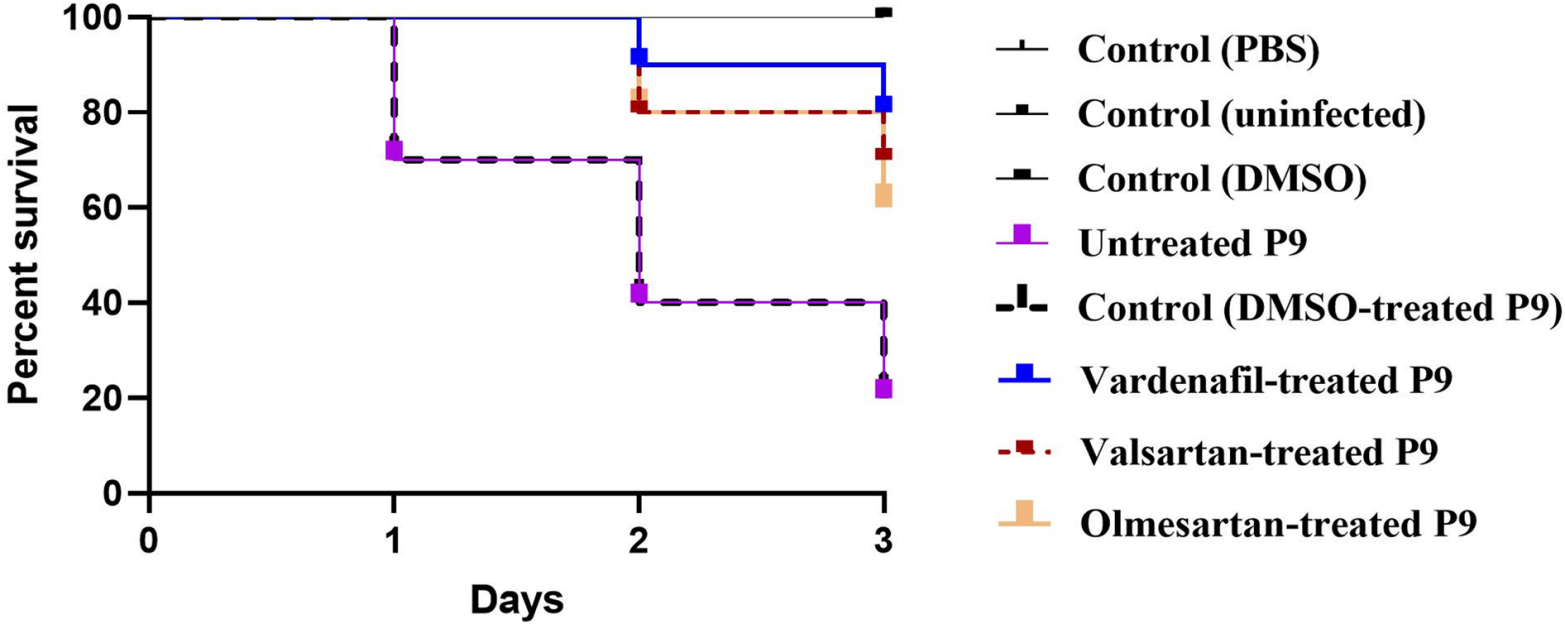
Protective effect of repurposed drugs in a murine infection model. Treatment with vardenafil, valsartan, and olmesartan significantly attenuated *P. aeruginosa* pathogenesis, resulting in survival rates of 80%, 70%, and 60%, respectively, compared to 20% survival in mice infected with untreated bacteria. The drugs were tested at their 1/8 MIC values (0.39 mg/mL for vardenafil and 0.2 mg/mL for valsartan and olmesartan). Mouse survival was analyzed using the Kaplan–Meier method, GraphPad Prism 8. **P9**: a highly virulent *P. aeruginosa* clinical isolate.

To evaluate the protective effects of the tested agents against the pathogenicity of *P. aeruginosa*, a mouse survival assay was conducted **(**Fig. 10**).** The drugs were tested at their 1/8 MIC values (0.39 mg/mL for vardenafil and 0.2 mg/mL for valsartan and olmesartan). All mice in the negative control groups showed 100% survival. In contrast, only 20% survival was detected in mice injected with untreated or DMSO-treated *P. aeruginosa* (positive control groups) at the end of the experiment. Notably, 80% of mice injected with vardenafil-treated *P. aeruginosa* remained alive. In the valsartan-treated group, 7 out of 10 mice (70%) survived, whereas four deaths (60% survival) were recorded in the group injected with olmesartan-treated *P. aeruginosa*.

### Molecular docking study

Protein models were validated using Procheck and ERRAT to ensure suitability for molecular docking. Ramachandran plot analysis revealed that 91.3%, 87.5%, and 88.8% of amino acid residues of LasR, PqsR, and RhlR, respectively, were located in the most favored regions, while 8.7%, 12.0%, and 10.9% of residues were found in additionally allowed regions, indicating the structural stability of the proteins (**Supplementary Fig. S2).** The ERRAT quality factors for LasR, PqsR, and RhlR were 99.84%, 90.77%, and 91.06%, respectively, confirming high-quality protein models^20^. Furthermore, the obtained root mean square deviation (RMSD) values of OHN (native ligand of LasR), MRD (native ligand of PqsA), HL4 (native ligand of RhlR) were 0.7, 0.94, and 0.79 Å, respectively **(Supplementary Tables S2–S4)**, confirming the accuracy and robustness of the docking protocol.

### Docking analysis at *P. aeruginosa* LasR QS receptor

**Fig. 11:**
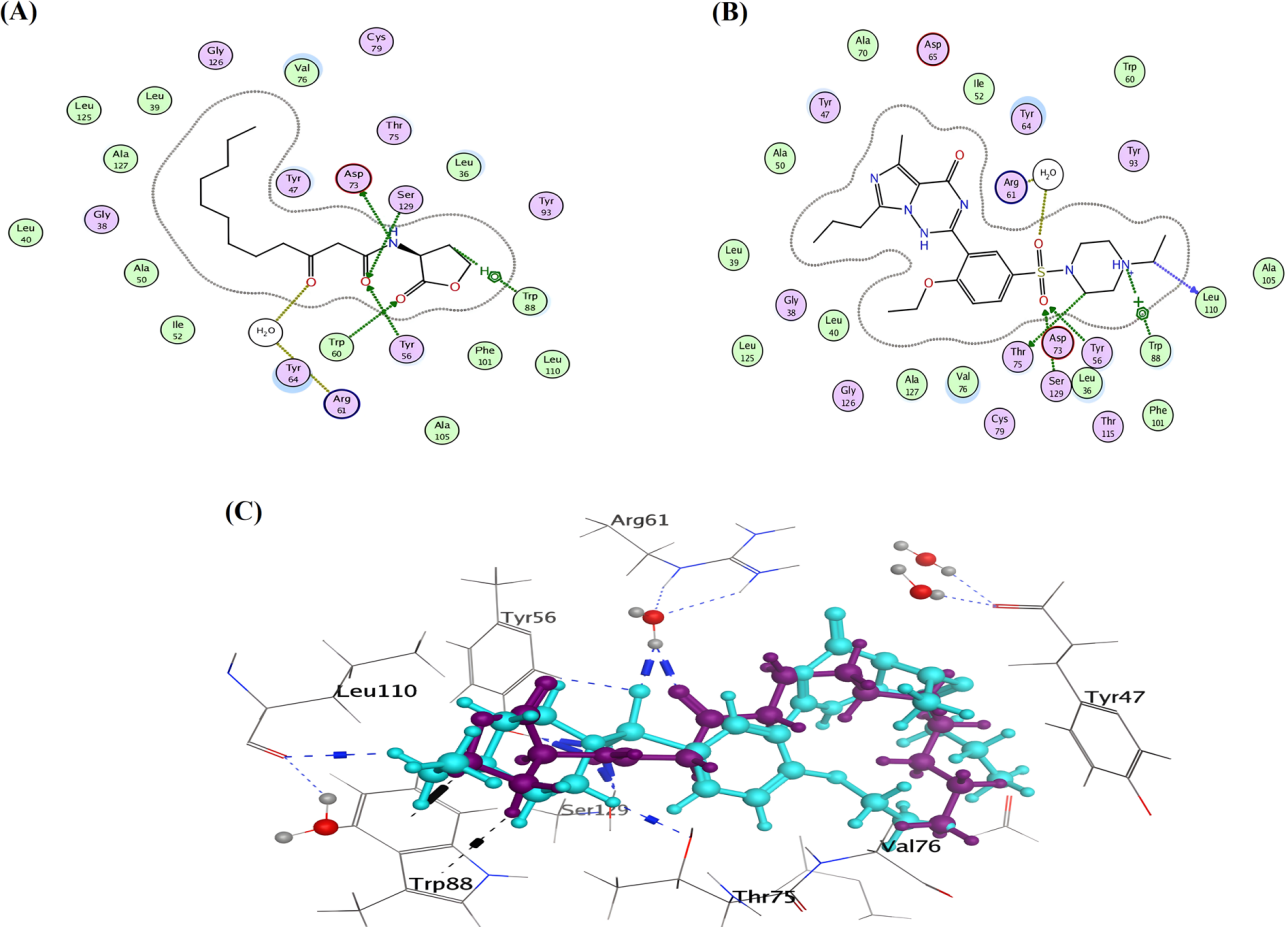
The molecular docking results of vardenafil and native ligand (OHN) at LasR receptor (PDB ID: 2UV0). **(A**,** B)** Two-dimensional interaction maps of the native ligand OHN (dark magenta) and vardenafil (cyan) within the LasR active site. **(C)** Three-dimensional overlay view of vardenafil and the reference ligand OHN within the LasR active site. For clarity, non-interacting residues were omitted. Hydrogen bonds are shown in blue, while π-interactions are shown in black.

Docking analysis demonstrated that vardenafil exhibited the highest binding affinity toward the LasR receptor, fitting appropriately within its active-site cavity with a docking score of − 7.49 kcal/mol compared with the native ligand (OHN), which showed a docking score of − 11.77 kcal/mol **(**Fig. 11; **Supplementary Table S2)**. Vardenafil formed four hydrogen bonds with Leu110, Thr75, Tyr56, and Ser129, in addition to a water-mediated hydrogen bonding with Arg61 and a cation–π interaction with Trp88. Notably, several key amino acid residues were shared between the LasR–vardenafil and LasR–OHN complexes, including Arg61, Tyr56, Ser129, and Trp88. In contrast, valsartan and olmesartan exhibited no significant binding interactions with LasR receptor **(Supplementary Table S2)**.

### Docking analysis at *P. aeruginosa* PqsR QS receptor

**Fig. 12:**
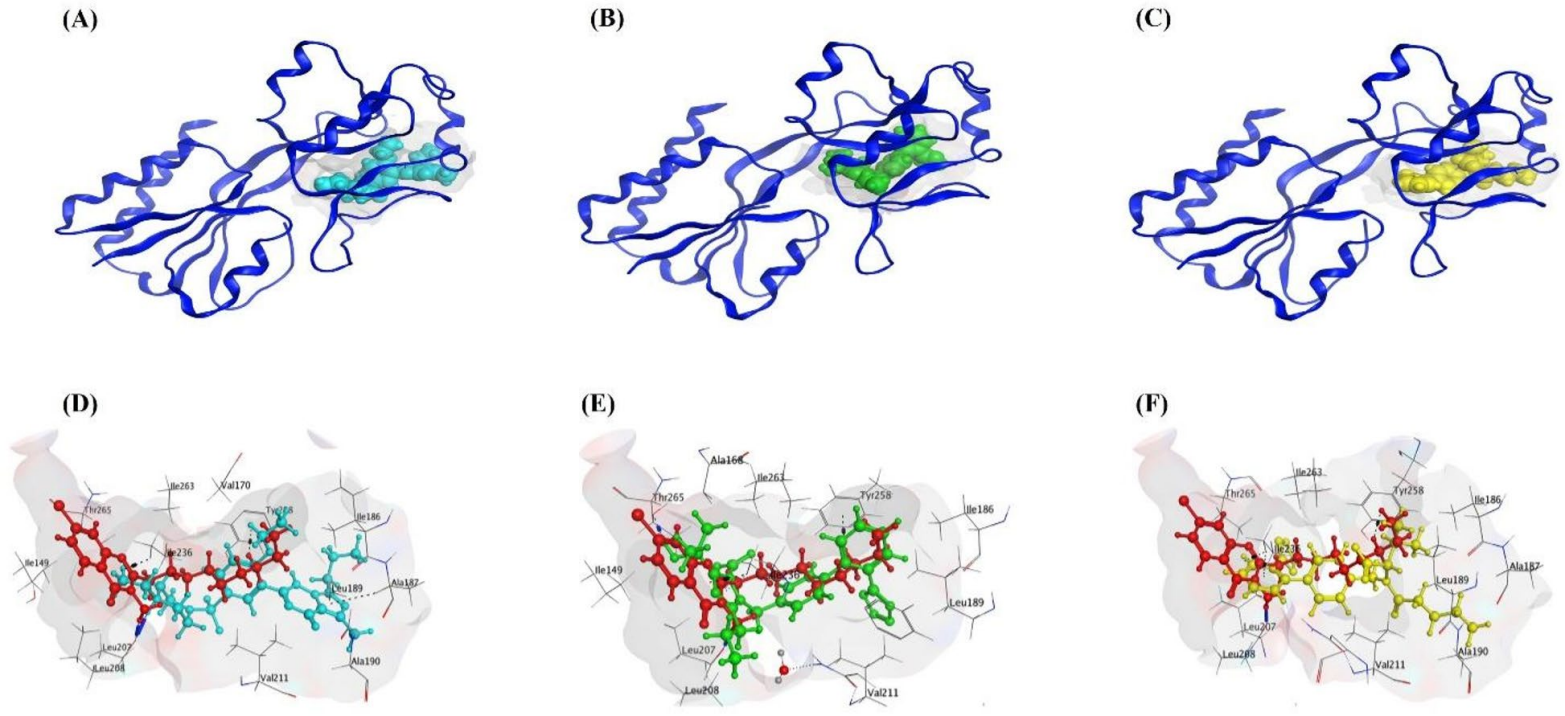
The molecular docking results of PqsR inhibitor, vardenafil, valsartan, and olmesartan at PqsR receptor (PDB ID: 4JVC). **(A–C)** 3D cartoon representations of vardenafil (cyan), olmesartan (green), and valsartan (yellow) within PqsR receptor. **(D–F)** Overlay of the tested drugs with the PqsR inhibitor (red) at the PqsR binding site. Non-interacting residues were excluded for clarity. Hydrogen bonds are indicated in blue, while π-interactions are represented in black.

Regarding binding with PqsR receptor, the PqsR competitive inhibitor (3-amino-7-chlorinated quinazolinone analogue)^21^, PqsR native ligand (MRD), and tested drugs were docked inside the PqsR active site with different scoring functions. Among the tested compounds, vardenafil demonstrated the highest binding affinity toward PqsR, with a docking score of − 6.77 kcal/mol, exceeding that of the PqsR inhibitor (− 6.59 kcal/mol) and the native ligand MRD (− 3.69 kcal/mol). It interacted with Leu207 by hydrogen bond and Tyr258 by π-π stacking interaction. The hydrogen bonding with Leu207 was common in vardenafil, PqsR inhibitor, and MRD, whereas both vardenafil and PqsR inhibitor shared a similar π interaction with Tyr258 within the same active site **(**Fig. 12; **Supplementary Fig. S3 and Table S3)**.

Olmesartan and valsartan demonstrated higher PqsR binding affinity of − 5.63 kcal/mol and − 5.32 kcal/mol, respectively, than the native ligand MRD (− 3.69 kcal/mol). Moreover, olmesartan and valsartan exhibited interaction patterns similar to those of the PqsR inhibitor, including a π interaction with Tyr258 and an *H*–π interaction with Ile236, respectively **(**Fig. 12; **Supplementary Fig. S3 and Table S3)**.

### Docking analysis at *P. aeruginosa* RhlR QS receptor

**Fig. 13:**
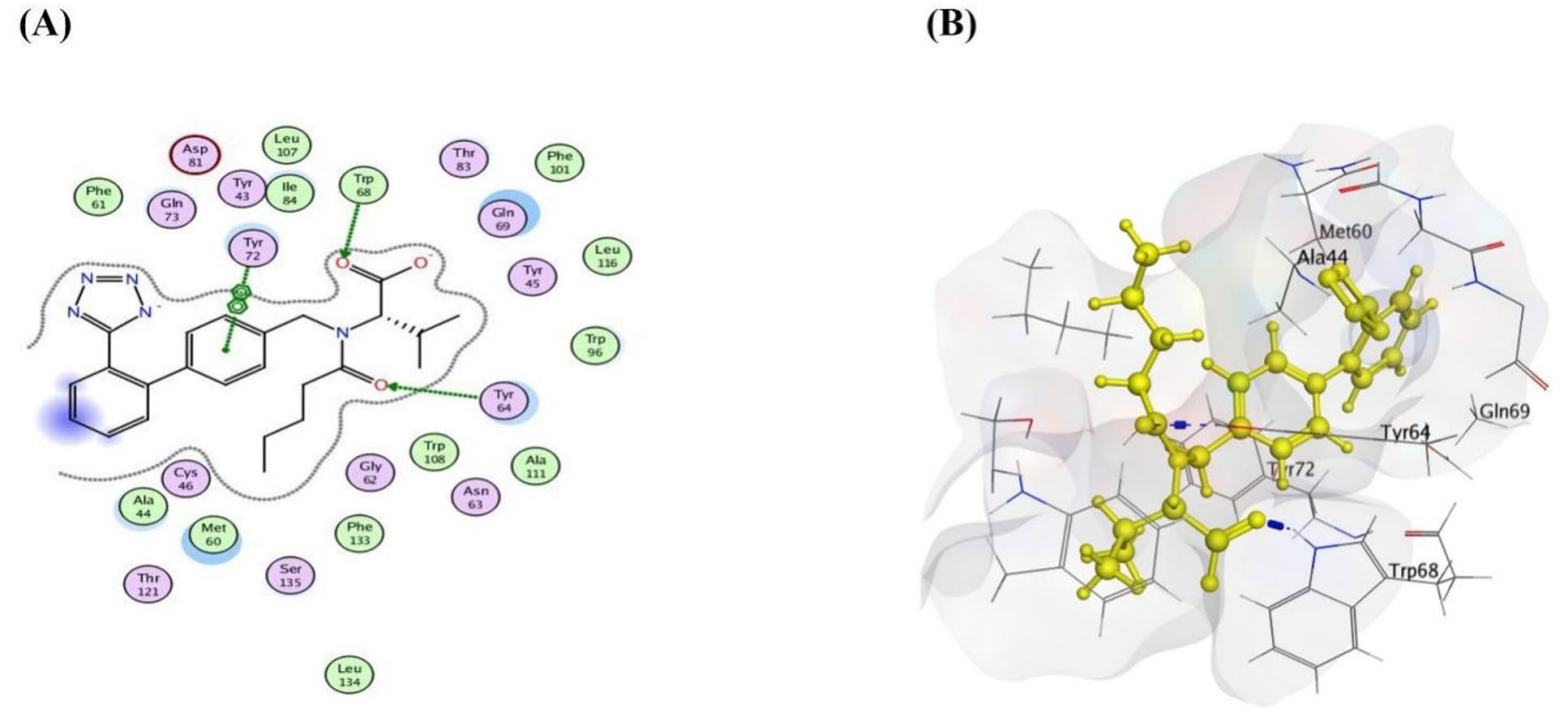
The molecular docking results of valsartan at RhlR receptor (PDB ID: 7R3H). **(A**,** B)** 2D and 3D ligand interactions of valsartan (yellow) with key residues of RhlR. Non-interacting aminoacids were deleted. Hydrogen bonds are shown in blue, and π interactions are shown in black.

Our docking results also revealed that valsartan has a proper orientation within the RhlR active site with a binding score of − 3.13 kcal/mol compared to native ligand (HL4), which exhibited a docking score of − 6.55 kcal/mol. Similar interactions with Trp68 and Trp64 (*H-*bonds) were detected in both valsartan and native ligand HL4 at the RhlR active site **(**Fig. 13; **Supplementary Table S4)**. In contrast, neither olmesartan nor vardenafil exhibited significant binding interactions with the RhlR receptor **(Supplementary Table S4).**

## Discussion

*Pseudomonas aeruginosa* is a major opportunistic pathogen associated with nosocomial infections, particularly in immunocompromised patients, including those with diabetes or cancer^22^. Its ability to cause diverse and invasive infections contributes to extended hospitalization and high mortality^23^. Additionally, the emergence of multi-drug resistance and persister strains has markedly restricted treatment strategies, representing a serious worldwide concern^24^.

In the present study, vardenafil, valsartan, and olmesartan were repurposed and evaluated as potential anti-virulence and antibiofilm agents against clinical isolates of *P. aeruginosa*. The tested compounds also exhibited antimicrobial activity against ten *P. aeruginosa* isolates **(Supplementary Table ****S1****)**. Consistent with these findings, a previous study reported that telmisartan achieved a lower MIC value of 62.5 µg/mL against *P. aeruginosa*^18^. Instead, the MIC range of vardenafil is similar to that of sildenafil, which inhibited *P. aeruginosa* growth at concentrations ranging from 3.12 to 6.25 mg/mL^15^.

*P. aeruginosa* pathogenicity depends on several virulence factors, including hemolysins, proteases, pyocyanin, lipases, and swarming motility, which are essential for infection establishment^25^. A scoring system based on counting isolates with ≥ 50% inhibition was applied to account for variability between the ten clinical isolates. Scores were assigned for each virulence factor and summed to compare the overall anti-virulence activity of the tested drugs. Notably, vardenafil achieved the highest total score, indicating its superior potential as anti-virulence agent, followed by valsartan, with olmesartan exhibiting the lowest effect.

In the present study, vardenafil exhibited a strong inhibitory effect on multiple virulence factors of *P. aeruginosa* **(**Figs. 3, 4, 5, 6 and 7**)**. These findings are consistent with previous reports showing comparable inhibition of hemolysin (92%) and swarming motility (74.79%) using sitagliptin^26^, as well as protease (87%) and pyocyanin (75%) inhibition by vitamin E against PAO1^27^. In addition, valsartan and olmesartan significantly reduced these virulence factors in *P. aeruginosa* isolates with comparable inhibition profiles **(**Figs. 3, 4, 5, 6 and 7**)**. These findings align with an Egyptian study reporting similar anti-virulence effects of candesartan, particularly against hemolysins (96.5%) and lipases (50%), with relatively lower protease inhibition (60%)^28^.

Biofilm formation represents a major barrier to effective antibiotic therapy and contributes to increased resistance^29^. Vardenafil exerted the highest antibiofilm activity against the ten isolates, followed by valsartan, then olmesartan **(**Fig. 8**)**. Comparable antibiofilm effects were recorded for thymoquinone (63%), naproxen (48–63%), and cilostazol (37.42%)^30–32^, whereas sildenafil and telmisartan exhibited higher antibiofilm activity of 87% and 89%, respectively^15,18^.

The promising phenotypic findings were further validated by quantitative reverse transcription PCR (qRT-PCR) analysis of QS-related genes (*lasR*, *lasI*, *pqsA*, *fliC*, *exoS*, and *pslA*) in the five *P. aeruginosa* isolates **(**Fig. 9**)**. Vardenafil significantly downregulated all tested genes, with the strongest effect observed on the Las QS system (*lasR* (up to 20-fold) > *lasI* (up to 11-fold)) **(**Fig. 9A **and B)**. Cilostazol, a PDE3 inhibitor, markedly suppressed QS gene expression, particularly *lasR* gene (99.5%)^30^, This could support the role of PDE inhibitors, including vardenafil, in disrupting QS and reducing bacterial virulence.

The *pqsA* gene was also downregulated by up to 7.7-fold **(**Fig. 9C**)**, reflecting inhibition of the PQS signaling pathway involved in virulence and biofilm formation^33^. The *exoS* gene encodes Exoenzyme S, a type III secreted toxin that plays a critical role in the virulence of *P. aeruginosa* by disrupting host cell signaling and cytoskeletal organization^34^. Vardenafil treatment resulted in up to 6.6-fold downregulation of *exoS* expression **(**Fig. 9E**)**, which may result from vardenafil-mediated suppression of QS regulators, particularly LasR, which is known to influence *exoS* expression^35^. Moderate downregulation of *fliC* and *pslA* suggested partial impairment of motility and biofilm matrix formation, respectively **(**Fig. 9D **and F).** Specifically, *fliC* encodes the flagellin subunit required for flagellar motility, while *pslA* is responsible for synthesizing a critical exopolysaccharide that stabilizes the biofilm matrix^36,37^.

Moreover, our qRT-PCR results demonstrated that valsartan and olmesartan treatment caused the most pronounced downregulation in *lasI* expression (up to 7.1 and 7.69-fold, respectively), with moderate effects on *lasR*, *fliC*, and *exoS* **(**Fig. 9**).** The marked suppression of *lasI* suggests that interference with autoinducer production may represent an important mechanism underlying the observed inhibition of QS-regulated virulence phenotypes^33,38^. Similarly, but to a lesser extent, Nazeih et al. reported that the antihypertensive drug glyceryl trinitrate significantly reduced the *lasI* (41.67%) and *lasR* (20.43%) expression at sub-MIC levels in *P. aeruginosa*^39^. In the present study, valsartan exhibited a stronger inhibitory effect on the *las* and *fliC* genes **(**Fig. 9**)**, indicating effective suppression of bacterial communication and motility-associated virulence traits^40,41^. On the other hand, olmesartan exerted a more pronounced downregulatory effect on the *pqsA* and *exoS* genes **(**Fig. 9C **and E)**, suggesting a preferential interference with PQS-mediated signaling and acute virulence mechanisms in *P. aeruginosa*^42^.

To further confirm the anti-virulence activity of tested drugs, the in vivo protection assay was conducted. Treatment with vardenafil, valsartan, and olmesartan markedly improved mouse survival **(**Fig. 10**).** Cilostazol and candesartan also showed improved survival and reduced pathogenicity in murine infection models following QS inhibition^19,30^. Consequently, a comprehensive molecular docking study was performed to assess the potential of the tested drugs to interact with key QS proteins in the bacterial strains **(Supplementary Tables S2–S4).** This could suggest possible anti-QS and anti-virulence activities of tested drugs.

Overall, these findings suggest that vardenafil may exert anti-QS activity. This effect may be also attributed to its lipophilic nature, which may facilitate penetration of the Gram-negative bacterial membrane and enhance interactions with intracellular QS components^43,44^. Another possible explanation is that vardenafil may indirectly affect intracellular signaling pathways linked to quorum sensing through modulation of cyclic nucleotide signaling. Consequently, alterations in second-messenger signaling can influence QS regulatory networks, including LasR^45,46^.

Furthermore, the superior anti-virulence activity of valsartan compared to olmesartan may be attributed to its stronger modulation of the Las system and its higher lipophilicity^47,48^. Also, the effects observed following ARBs treatment may be associated with drug-induced metabolic and stress adaptations in bacterial cells^49,50^. Such responses can reduce energy-demanding biosynthetic processes, including the production of quorum-sensing autoinducers mediated by *lasI*^51^.

Notably, the sub-MIC concentrations of all tested drugs exceeded the concentrations required to achieve therapeutic tissue levels following oral administration^52^. Therefore, systemic use for anti-virulence purposes may be limited. Nevertheless, *P. aeruginosa* is a major causative agent of several localized infections, including burn and chronic wound infections, diabetic foot infections, and ocular infections^53^. Hence, these drugs may be more suitably considered for topical application using formulations with improved lipophilic properties^54–56^. Such formulations could potentially be employed as adjuncts to conventional antibiotics in the treatment of aggressive *P. aeruginosa* infections.

The present study did not include QS-deficient mutant strains or established QS inhibitors/quorum-quenching enzymes as reference controls. In addition, only swarming motility was evaluated as a representative QS-regulated phenotype. Future studies incorporating these controls and examining additional motility phenotypes, including swimming and twitching motilities, would provide further mechanistic insight into the anti-virulence activity of the tested compounds.

Collectively, these findings highlight the potential of vardenafil, valsartan, and olmesartan as promising anti-virulence agents that selectively attenuate bacterial pathogenicity without inhibiting bacterial growth. Their QS-targeted activity suggests possible utility in topical applications as adjunctive therapeutic strategies against *P. aeruginosa* infections, warranting further investigation.

## Materials and methods

### Ethics statement

All methods used in this study were conducted in accordance with the relevant institutional guidelines and local regulatory requirements. The requirement for approval and informed consent for the use of clinical *P. aeruginosa* isolates was waived by the Ethical Committee of the Faculty of Medicine, Zagazig University, Egypt (IRB#: 11272-8-11-2023), as the isolates were obtained from routine hospital laboratory collections and were fully anonymized prior to analysis. All experiments involving clinical isolates were performed in accordance with applicable institutional and regulatory guidelines. Animal experimental procedures were reviewed and approved by the Institutional Animal Care and Use Committee of Zagazig University, Egypt (ZU-IACUC; Approval No.: ZUIACUC/3/F/377/2023) and were conducted in compliance with relevant ethical standards and regulations.

### Bacterial strains, identification, and chemicals

Ten clinical *P. aeruginosa* isolates were selected from a total of 50 isolates based on their elevated production of hemolysin, protease, lipase, and pyocyanin, as well as their swarming motility and high biofilm formation. Clinical isolates (P1–P10) were obtained from the culture collections of Zagazig University Hospitals, Egypt. Collected specimens were inoculated on MacConkey agar (Oxoid, UK) and incubated at 37 °C for 24 h. Non-lactose-fermenting pale colonies on MacConkey agar were identified as *P. aeruginosa* based on standard microbiological criteria, including Gram staining, pigment production on cetrimide agar (Oxoid, UK), motility, citrate utilization testing, and growth at 42 °C^57^. Vardenafil, valsartan, and olmesartan were obtained from Sigma Chemical Company, St. Louis, Mo, USA. DMSO controls were incorporated throughout all experimental procedures.

### The effect of vardenafil, valsartan, and olmesartan on *P. aeruginosa* growth

The MICs of tested compounds were evaluated against the tested isolates using the broth microdilution method according to the Clinical Laboratory and Standards Institute guidelines (CLSI, 2024)^58^. Vardenafil, valsartan, and olmesartan were dissolved using DMSO as a solvent to prepare stock solutions of 50 mg/mL. Two-fold serial dilutions of each compound were prepared in Mueller–Hinton broth (MHB, Oxoid, UK) using 96-well microtiter plates, yielding final concentrations ranging from 25,000 to 48.83 µg/mL. Overnight cultures of the isolates were diluted in MHB to obtain a bacterial suspension adjusted to 1 × 10⁶ CFU/mL. Incubation was carried out at 37 °C for 24 h. Based on the determined MIC values, sub-inhibitory concentrations (1/8 MIC) were selected for all subsequent experiments. These concentrations ranged from 0.39 to 0.78 mg/mL for vardenafil and from 0.2 to 0.39 mg/mL for valsartan and olmesartan.

The impact of the tested drugs on the growth of ten *P. aeruginosa* isolates was evaluated^32^. Briefly, bacterial cultures, either untreated or treated with the tested drugs at 1/8 MIC, were assessed for growth using viable cell counts and optical density measurements at 600 nm.

### Hemolysin inhibition assay

The hemolytic activity of *P. aeruginosa* in the presence and absence of the tested drugs was evaluated^59^. Briefly, culture supernatants were harvested from bacterial cultures grown with or without sub-MIC concentrations of tested drugs. Equal volumes of the supernatants and a 2% suspension of sheep erythrocytes were mixed and incubated at 37 °C for 2 h. The mixtures were then centrifuged, and the absorbance of the resulting supernatants was measured at 540 nm. Unhemolyzed erythrocytes served as the negative control, while completely hemolyzed erythrocytes were used as the positive control.

### Protease inhibition assay (modified skimmed milk broth)

Overnight *P. aeruginosa* cultures grown in MHB in the presence or absence of the tested drugs (1/8 MIC) were centrifuged to obtain cell-free supernatants. The proteolytic activity was assessed by incubating 500 µL of culture supernatant with 1 mL of 1.25% skim milk at 37 °C for 1 h, followed by measuring the reduction in optical density at 600 nm^60^.

### Pyocyanin inhibition assay

The inhibitory effects of the tested drugs on pyocyanin production by *P. aeruginosa* were assessed as previously described^61^. Briefly, *P. aeruginosa* isolates were cultured overnight in Luria–Bertani broth (LBB, Oxoid, UK), and then adjusted to an optical density of 0.4 at 600 nm. Fresh LBB tubes (1 mL), either treated with the tested drugs at 1/8 MIC or untreated, were inoculated with 10 µL of the prepared bacterial suspension and incubated at 37 °C for 48 h. After incubation, culture supernatants were harvested by centrifugation, and pyocyanin production was quantified by measuring absorbance at 691 nm.

### Lipase inhibition assay by Tween 80 opacity test

Overnight *P. aeruginosa* cultures grown in MHB were adjusted to a turbidity equivalent to the 0.5 McFarland standard. Subsequently, a 2 µL aliquot of the prepared bacterial suspension was spot-inoculated onto Tween 80 agar plates containing 0.1 g CaCl₂, 15 g agar, 5 g NaCl, and 10 g Bacto Peptone per liter of distilled water. Tween 80 was incorporated into the medium at a final concentration of 1% (v/v). The plates were prepared with or without sub-inhibitory concentrations of the tested drugs and incubated at 37 °C for 72 h. Lipase activity was then evaluated by measuring the diameter of the opaque halo surrounding the inoculation site^62^.

### Swarming motility inhibition assay

The impact of the tested drugs on swarming motility was evaluated using LB agar plates (0.5% agar) supplemented with the tested drugs at 1/8 MIC, alongside control plates. Two microliters of diluted overnight cultures were surface-inoculated on the plates. Following incubation at 37 °C for 18 h, swarming zone diameters were measured and compared^63^.

### Biofilm inhibition assay

The antibiofilm activity of the tested drugs was evaluated using a modified microtiter plate assay^64^. Overnight cultures were diluted in Tryptone Soya Broth (TSB, Oxoid, UK) to 1 × 10⁶ CFU/mL, and 100 µL of the bacterial suspension was added to 96-well plates containing TSB alone or TSB supplemented with the tested drugs at 1/8 MIC. After incubation at 37 °C for 24 h, wells were washed, and adherent biofilms were fixed with 99% methanol, stained with 1% crystal violet, and solubilized with 33% glacial acetic acid. Biofilm formation was quantified by measuring absorbance at 590 nm. All experiments were performed in triplicate.

### Effect of different sub-MIC concentrations of vardenafil on virulence factor production

To further investigate the concentration-dependent anti-virulence activity of vardenafil, additional sub-MIC concentrations corresponding to 1/4 and 1/2 MIC were evaluated alongside 1/8 MIC against five representative *P. aeruginosa* isolates (P1, P3, P7, P9, and P10). The effects of the different concentrations on the tested virulence factors were assessed using the same experimental procedures described above.

### Effect of vardenafil, valsartan, and olmesartan on the expression of QS genes

From the ten examined isolates, the five isolates (P1, P3, P7, P9, and P10) exhibiting the highest phenotypic virulence and biofilm formation were selected to evaluate the effects of the tested drugs on QS gene expression. The sub-inhibitory concentrations (1/8 MIC) used in this experiment ranged from 0.39 to 0.78 mg/mL for vardenafil and from 0.2 to 0.39 mg/mL for valsartan, while a concentration of 0.2 mg/mL was used for olmesartan.

Total RNA was isolated from *P. aeruginosa* cultures, either untreated or treated with the tested drugs at sub-inhibitory concentrations, as previously described^65^. The extracted RNA was reverse-transcribed into cDNA, and the expression levels of QS genes were quantified using qRT-PCR. The gene expression was normalized to the house keeping gene *ropD*, and the primer sequences used are presented in Table 1. Relative changes in gene expression were calculated using the ^ΔΔ^Ct method^66^.

**Table 1:** List of the used primers in the study.

Gene name	Type	Nucleotide Sequence (5’ to 3’)	References
*lasI*	F	CGCACATCTGGGAACTCA	^72^
	R	CGGCACGGATCATCATCT	
*lasR*	F	CTGTGGATGCTCAAGGACTAC	^72^
	R	AACTGGTCTTGCCGATGG	
*pqsA*	F	GACCGGCTGTATTCGATTC	^72^
	R	GCTGAACCAGGGAAAGAAC	
*fliC*	F	GCTTCGACAACACCATCAAC	^73^
	R	AGCACCTGGTTCTTGGTCAG	
*exoS*	F	CCATCACTTCGGCGTCACT	^72^
	R	GAGAGCGAGGTCAGCAGAG	
*pslA*	F	TCCCTACCTCAGCAGCAAGC	^74^
	R	TGTTGTAGCCGTAGCGTTTCTG	
*ropD*	F	CGAACTGCTTGCCGACTT	^72^
	R	GCGAGAGCCTCAAGGATAC	

F: forward, R: reverse.

### Mice survival assay

One *P. aeruginosa* isolate (P9) was selected for the in vivo assay. This isolate was chosen based on its strong virulence-associated phenotypes, and favorable response to all tested drugs. It was also classified as an extensively drug-resistant strain (XDR). The selected isolate P9 was exposed to sub-inhibitory concentrations (1/8 MIC) of the tested drugs, corresponding to 0.39 mg/mL for vardenafil and 0.2 mg/mL for valsartan and olmesartan.

The protective potential of the tested drugs against *P. aeruginosa* pathogenesis was evaluated in a murine survival model^67^. Overnight cultures of *P. aeruginosa* were prepared in LBB with or without 1/8 MIC of the tested agents, then diluted in phosphate-buffered saline (PBS) to a final concentration of 2.5 × 10⁷ CFU/mL. Three-week-old female albino mice (*Mus musculus*) were randomly assigned to eight groups of ten mice each. Groups 1–3 were injected intraperitoneally with 100 µL of bacteria treated with vardenafil, valsartan, or olmesartan, respectively. Group 4 received untreated bacteria, while group 5 received DMSO-treated bacteria. Groups 6–8 served as negative controls: group 6 received PBS, group 7 received DMSO, and group 8 remained uninoculated. Mice were maintained under standard conditions, and survival was monitored daily for three days. Survival curves were generated using the Kaplan–Meier method in GraphPad Prism 8.

### Molecular docking study

Docking studies were performed for the most stable conformers of vardenafil, valsartan, and olmesartan against various *P. aeruginosa* QS biotargets to assess their potential anti-virulence activity. The selected biological targets included: (I) LasR receptor (Protein data bank (PDB) ID: 2UVO)^68^, (II) PqsR receptor (PDB ID: 4JVC)^69^, and (III) RhlR receptor (PDB ID: 7R3H)^70^. Ligand molecular structures were drawn using ChemDraw Professional 12.0. Each ligand was then protonated in three dimensions, assigned partial charges, energy-minimized, and saved. Target proteins were prepared by removing non-functional chain sequences, leaving only the chains implicated in protein function. Finally, the prepared ligands were loaded and docked into the active sites of LasR, PqsR, and RhlR proteins.

Protein quality was validated using the Procheck and ERRAT web servers (https://saves.mbi.ucla.edu/). Ramachandran plot analysis was provided by Procheck to evaluate the overall stereochemical quality of the proteins, while ERRAT assessed the amino acid environment^20^. In addition, the RMSD was determined to quantify the structural agreement between the co-crystallized and re-docked ligand conformations. Docking was performed by generating 30 conformations per ligand, and the most energetically favorable conformers with RMSD values < 2 Å were selected for further analysis^71^.

### Statistical analysis

GraphPad Prism 8 was used for statistical analysis. The effects of the tested agents on virulence factor expression were evaluated using one-way ANOVA followed by Dunnett’s multiple comparison test. The impact of the tested agents on bacterial growth was analyzed using paired *t*-test. A *p*-value < 0.05 was considered statistically significant.

## Supplementary Information

Below is the link to the electronic supplementary material.

Supplementary Material 1

## Data Availability

All data generated or analyzed during this study are included in this published article and its supplementary information files.
