# Supplementary material for: Disarming Pseudomonas aeruginosa: repurposing vardenafil, valsartan, and olmesartan as anti-virulence and antibiofilm agents
Source: Sci Rep. 2026 Aug 7;16:24455. doi: 10.1038/s41598-026-64238-9 (PMC13451190; doi:10.1038/s41598-026-64238-9)
Supplement: Supplementary file 1 [file 41598_2026_64238_MOESM1_ESM.docx]

**Supplementary Information**

**Disarming *Pseudomonas aeruginosa*: Repurposing Vardenafil, Valsartan, and Olmesartan as Anti-virulence and Antibiofilm Agents.**

**Aya A. Mahfouz ^a, b^, Hisham A. Abbas ^a^,** [**Nehal Yousef**](https://link.springer.com/article/10.1186/s12866-022-02515-z?utm_source=chatgpt.com#auth-Nehal-Yousef-Aff1) **^a^, Abdelaziz Elgaml ^b, c *^**

^a^ Department of Microbiology and Immunology, Faculty of Pharmacy, Zagazig University, Zagazig 44519, Egypt

^b^ Department of Microbiology and Immunology, Faculty of Pharmacy, Horus University-Egypt, New Damietta 34518, Egypt*.*

^c^ Department of Microbiology and Immunology, Faculty of Pharmacy, Mansoura University, Mansoura 35516, Egypt*.*

* Corresponding author: Abdelaziz Elgaml

E-mail addresses: [elgamel3a@mans.edu.eg](mailto:elgamel3a@mans.edu.eg)

**Table S1.** Minimum inhibitory concentrations of vardenafil, valsartan, and olmesartan against the tested *P. aeruginosa* isolates.

**Isolate no.:** isolate number, **MIC:** Minimum inhibitory concentrations.

| **Isolate no.** | **MIC of vardenafil (mg/mL)** | **MIC of valsartan (mg/mL)** | **MIC of olmesartan (mg/mL)** |
| --- | --- | --- | --- |
| **P1** | 6.25 | 1.56 | 1.56 |
| **P2** | 6.25 | 3.12 | 1.56 |
| **P3** | 3.12 | 1.56 | 1.56 |
| **P4** | 6.25 | 3.12 | 3.12 |
| **P5** | 6.25 | 3.12 | 3.12 |
| **P6** | 6.25 | 1.56 | 1.56 |
| **P7** | 3.12 | 1.56 | 1.56 |
| **P8** | 3.12 | 3.12 | 1.56 |
| **P9** | 3.12 | 1.56 | 1.56 |
| **P10** | 6.25 | 3.12 | 1.56 |

**Table S2.** Docking results for native ligand (OHN), vardenafil, valsartan, and olmesartan binding at LasR protein (PDB ID: 2UV0).

| **Protein** | **Ligand/Drug** | **Docking binding energy (kcal/mol)** | **RMSD (Å)** | **Interactions (no. of bonds)** |
| --- | --- | --- | --- | --- |
| **LasR** | **Native ligand (OHN)** | -11.77 | 0.7 | **Hydrogen bonds—**  Agr61 (water-mediated) (1), Tyr56 (1), Ser129 (1), Trp60 (1), Asp73 (2)  ***H*–pi interaction—**  Trp88 (1)  **Hydrophobic interactions—**  Tyr64, Leu36, Val76, Tyr47 |
|  | **Vardenafil** | -7.49 | 1.9 | **Hydrogen bonds—**  Agr61 (water-mediated) (1), Tyr56 (1), Ser129 (1), Leu110 (1), Thr75 (1)  **cation–pi interaction—**  Trp88 (1)  **Hydrophobic interactions—**  Tyr64, Leu36, Val76, Tyr47 |
|  | **Valsartan** | -2.77 | 1.59 | **Hydrogen bonds—**  Tyr56 (1), Asp73 (1)  **pi–pi stacking—**  Trp88 (1)  **Hydrophobic interactions—**  Tyr64, Val76, Tyr47 |
|  | **Olmesartan** | -1.62 | 1.97 | **Hydrogen bonds—**  Tyr56 (1)  **pi–pi stacking—**  Trp88 (1)  **Hydrophobic interactions—**  Tyr64, Leu36, Val76, Tyr47 |

**RMSD:** root mean square deviation.

**Table S3.** Docking results for native ligand (MRD), PqsR inhibitor, vardenafil, valsartan, and olmesartan-binding at PqsR receptor (PDB ID: 4JVC).

| **Protein** | **Ligand/Drug** | **Docking binding energy (kcal/mol)** | **RMSD**  **(Å)** | **Interactions** |
| --- | --- | --- | --- | --- |
| **PqsR** | **Vardenafil** | -6.77 | 1.6 | **Hydrogen bond—**  Leu207  **pi–pi interactions—**  Tyr258  **Hydrophobic interactions—**  Leu207, Tyr258, Ile236, Ile263, Val211, Leu189, Ala187, Ile186, Ala190 |
|  | **PqsR inhibitor** | -6.59 | 0.86 | **Hydrogen bond—**  Leu207  ***H*–pi interactions—**  Tyr258, Ile236  **Hydrophobic interactions—**  Leu207, Tyr258, Ile236, Ile263, Ala168, Thr265, Leu208, Ile149, Val211, Leu189 |
|  | **Olmesartan** | -5.63 | 1.78 | **Hydrogen bond—**  Thr265  **pi–pi interaction—**  Tyr258  **Hydrophobic interactions—**  Leu207, Tyr258, Ile236, Ile263, Ala168, Thr265, Leu208, Ile149, Val211, Leu189 |
|  | **Valsartan** | -5.31 | 1.97 | ***H*–pi interaction—**  Ile236  **Hydrophobic interactions—**  Leu207, Tyr258, Ile236, Ile263, Thr265, Leu208, Ile149, Val211, Leu186, Ala187, Ala190 |
|  | **Native ligand (MRD)** | -3.69 | 0.94 | **Hydrogen bond—**  Leu207  **Hydrophobic interactions—**  Leu207, Ile236, Ile263, Thr265, Leu208 |

**RMSD:** root mean square deviation.

**Table S4.** Docking results for native ligand (HL4), vardenafil, valsartan, and olmesartan binding at RhlR protein (PDB ID: 7R3H).

| **Protein** | **Ligand/Drug** | **Docking binding energy (kcal/mol)** | **RMSD**  **(Å)** | **Interactions (no of bonds)** |
| --- | --- | --- | --- | --- |
| **RhlR** | **Native ligand (HL4)** | -6.46 | 0.79 | **Hydrogen bonds—**  Trp68 (1), Tyr64 (1), Asp81(1)  ***H*–pi interaction—**  Trp96 (1)  **Hydrophobic interactions—**  Tyr64, Trp96, Tyr72, Ile84, Ala44 |
|  | **Valsartan** | -3.13 | 1.94 | **Hydrogen bonds—**  Trp68 (1), Tyr64 (1)  **pi–pi interaction—**  Tyr72 (1)  **Hydrophobic interactions—**  Tyr64, Tyr72, Ala44, Gln69, Met60 |
|  | **Olmesartan** | -2.65 | 1.67 | **Hydrogen bonds—**  Trp68 (1)  **pi interactions—**  Tyr72 (1), Tyr64 (2)  **Hydrophobic interactions—**  Tyr64, Tyr72, Ile84, Ala44, Gln69 |
|  | **Vardenafil** | -1.7 | 1.75 | **Hydrogen bonds—**  Trp68 (1), Phe61 (1)  ***H*–pi interactions—**  Trp72 (1), Met60 (1)  **Hydrophobic interactions—**  Tyr64, Tyr72, Ala44, Gln69, Met60 |

**RMSD:** root mean square deviation.

|   **(b)**  **(c)**  **(a)** |   **(d)** |
| --- | --- |
|   **(e)** |   **(f)** |
|  |  |
|  |  |

**Fig. S1.** Concentration-dependent inhibition of virulence factors by vardenafil. The effects of vardenafil at 1/8, 1/4, and 1/2 MIC on **(a)** hemolysin production, **(b)** protease activity, **(c)** pyocyanin production, **(d)** lipase activity, **(e)** swarming motility, and **(f)** biofilm formation were assessed in five representative *P. aeruginosa* isolates (P1, P3, P7, P9, and P10). Results are expressed as percentage inhibition relative to the untreated control and presented as mean ± SD of three independent experiments.

Ramachandran plots to evaluate the overall stereochemical quality of LasR, PqsR, and RhlR proteins.

**(b)**

|   **(a)**  **(c)** |  | |
| --- | --- | --- |
|  |  |  |

**Fig. S2.** Ramachandran plots of the three-dimensional models of **(a)** LasR (PDB ID: 2UV0), **(b)** PqsR (PDB ID: 4JVC), and **(c)** RhlR (PDB ID: 7R3H). The most favored regions (A, B, L) are colored red, additional allowed (a, b, l, p) are colored yellow, generously allowed (~a, ~b, ~l, ~p) are colored light yellow and disallowed regions are indicated as white fields. **SER:** Serine. **ASN:** Asparagine. **CYS:** Cysteine. **HIS:** Histidine.

Docking analysis of native ligand, reference PqsR inhibitor, and repurposed drugs (vardenafil, valsartan, and olmesartan) within the PqsR receptor.

|   **(a)** |   **(b)** |
| --- | --- |
|   **(c)** |   **(d)** |
| **(e)** |  |

**Fig. S3.** The molecular docking results of native ligand (MRD), PqsR inhibitor, vardenafil, valsartan, and olmesartan at PqsR receptor (PDB ID: 4JVC). **(a-e)** 2D representations of MRD, PqsR inhibitor, vardenafil, valsartan, and olmesartan in the active site of PqsR.
